# Effect of Dolutegravir regimen against other regimens on some hematological parameters, CD4 count and viral load of people living with HIV infection in South Eastern Nigeria

**DOI:** 10.1097/MD.0000000000035910

**Published:** 2023-11-24

**Authors:** Stella N. Echefu, Joseph E. Udosen, Euphoria C. Akwiwu, Josephine O. Akpotuzor, Emmanuel Ifeanyi Obeagu

**Affiliations:** a Alex Ekwume Federal University Teaching Hospital Abakaliki, Ebonyi State, Nigeria; b Department of Surgery, University of Calabar, Nigeria; c Department of Haematology and Blood Transfusion Science, University of Calabar, Nigeria; d Department of Medical Laboratory Science, Kampala International University, Uganda.

**Keywords:** AIDS, CD4 count, Dolutegravir, HIV, PLWHIV, viral load suppression

## Abstract

**Background:**

Appropriate usage of highly active antiretroviral therapy (HAART) suppresses human immunodeficiency virus (HIV) replication. One of such HAART is dolutegravir (DTG) containing regimen which Nigeria included in her national protocol, as the preferred first-line option, with particularly fixed dose combination of tenofovir/lamivudine/dolutegravir (TLD) in 2018.

**Aim:**

To access the impact of this regimen as against other regimens on some hematological parameters as well as cluster of differentiation 4 (CD4) count and viral load on people living with HIV infection

**Methods:**

The study site is a health facility center supported by President Emergency Plan for acquired immunodeficiency syndrome (AIDS) Relief where people living with HIV infection (PLWHIV) visit for their routine management in Abakaliki, Ebonyi State. A hundred and twenty-two (122) subjects participated, 58 PLWHIV and 64 control subjects. CD4 + count by partec cyflow auto analyzer, while the Viral load assay was by Roche COBAS Ampriplep/COBAS TaqMan molecular systems. Full blood count determination was by Sysmex XE-2100 hematology auto analyzer, while the detection of antibody to HAART was by Petz and direct Coombs tests.

**Results:**

Mean values of hemoglobin (Hb), Total white cell count, Lymphocytes, Monocytes and CD4 + counts of people living with HIV infection (PLWHIV) were significantly (*P* = .0001) lower than the control subjects. The Hb level of PLWHIV on Efavirenz combination (TDF/3TC/EFV) are comparable 123 ± 32g/l with those on Ritonavir combination (TDF/3TC/LPV/R) 136 ± 16g/l and Dolutegravir (TLD)134 ± 20.0g/l (*P* = .307). On the other hand, total white cell count (4.55 ± 1.99 × 10^9^/L) of those on Efavirenz combination (TDF/3TC/EFV) and Dolutegravir (TLD) (4.53 ± 1.31 × 10^9^/L) were significantly higher than those on Ritonavir combination (TDF/3TC/LPV/R) (4.09 ± 1.15 × 10^9^/L). The Viral Load of PLWHIV on Dolutegravir (TLD) was significantly lower 171.57 ± 4.56 copies/mL than those on Efavirenz combination (TDF/3TC/EFV) (86,395.91 ± 27,476.57copies/mL) and Ritonavir combination (TDF/3TC/LPV/R) (81,188.83 ± 13,393.47 copies/mL), respectively.

**Conclusion:**

Some hematological parameters (such as Hb, total white cell counts and CD4 + count) were lower in people living with HIV than values seen in control group. The 3 regimens used in the management of HIV infection in the locality revealed comparable Packed cell volume and Hemoglobin levels. Total white cell count of those on Efavirenz and DTG is comparable with higher values than those on Ritonavir.

## 1. Introduction

Acquired Immunodeficiency Syndrome (AIDS) is a disease of human immune system that is characterized cytologically by reduction in the numbers of cluster of differentiation 4 (CD4) + bearing helper T cells as a result of infection by human immunodeficiency virus.^[1]^ It is characterized by immunosuppression that leads to opportunistic infection, secondary neoplasm and neurological manifestations.^[2]^ Human immunodeficiency virus (HIV) can infect many tissues but the 2 major targets are the immune system and the central nervous system.^[3]^ In 2020 HIV global statistics report has it that 37.7 million people are living with HIV and that,1.5 million people became newly infected with the virus while 680 000 people had died from AIDS-related illnesses.^[4]^ As of 2021, 27.5 million (73%) people had access to antiretroviral therapy.^[5]^

Appropriate use of HAART suppresses HIV replication but reports have shown some adverse effects of HAARTs to include kidney injury, decreased bone mineral density by nucleoside/nucleotide reverse transcriptase inhibitors e.g. Tenofovir, psychiatric and CNS disturbances by non-nucleoside/ reverse transcriptase inhibitors e.g. Efavirenz, creatinine glomerular filtration rate impairment as well as drug interactions with other medications by Integrase inhibitors such as dolutegravir (DTG)^[6–9]^ As at the end of 2017, Nigeria included DTG containing regimens in her national protocol, as the preferred first-line option, with particular fixed dose combination of tenofovir/lamivudine/dolutegravir (TLD).^[10]^ In Nigeria as at 2021,86% of the people living with HIV on HAART were virally suppressed.^[11]^ In another study, a rate of 75.91% was also reported among PLWHIV.^[12,13]^ Therefore, the aim/objective of the study is to evaluate the full blood count parameters, CD4 count, viral load and anti-drug-antibody production in people living with HIV infection on DTG as against other regimens in other to assess its impact. It is believed that the findings from this work will serve as baseline information on the use of DTG in the management of HIV infection in the locality as there is paucity of information in this regard from this part of the country.

## 2. Materials and methods

### 2.1. Study design

The study was a cross sectional, Case control involving people living with HIV who had strictly adhered to their drug regimen for over 1 year before the study.

### 2.2. Sample size

Sample size was calculated using precision rates for a finite population (Kothari, 2004) using the formula


$$\begin{array}{l} Minimum\;sample\;size\left(N\right)=Z^{2}pqN \end{array}$$

$$\begin{array}{l} e^{2}\left(N-1\right)+Z^{2}pq \end{array}$$

where N = population size = 2176,947 (NPC, 2006);

p = prevalence of HIV in Ebonyi state = 0.8%= (0.008) (NACA, 2019);

q = (1—p) = (1–0.008);

e = Acceptable level of error = 0.05;

Z = standard variant at 95% Confidence Interval (CI) = 1.96.


$$\begin{array}{l} \begin{array}{lll} & Hence,\;\left(n\right)=\frac{{\left(1.96\right)}^{2}\times \left(0.008\right)\times \left(1-.008\right)\times 2,176,947}{{\left(0.05\right)}^{2}\left(2,176,\;947-1\right)+{\left(1.96\right)}^{2}\times \left(0.008\right)\times \left(10.008\right)}\; & \;\;\;\;\;=66368.45/5442.40=12.19.\; \end{array} \end{array}$$

Therefore, n = 12. However, 58 subjects who showed willingness, as well as been on treatment adherence and consented were recruited in other to have enough population to make statistical inferential judgment.

### 2.3. Study area

The study was carried out on HIV subjects attending clinic in Alex Ekwueme Federal Teaching Hospital Abakaliki, Ebonyi State. The health facility is a known center supported by President Emergency Plan for AIDS Relief where people living with HIV infection visits for their routine management.

### 2.4. Subjects

A hundred and twenty-two (122) subjects participated in the study consisting of 58 PLWHIV and 64 control subjects. The control subjects were drawn from the general population consisting of staff and students of Ebonyi State University, Public and State Civil Servants etc using random sampling.

The inclusion criteria for the PLWHIV are that they must been enrolled at the facility for 1 year and above, must have been on any of the HAART regimen combination, must have adhered to the regimen for over 1 year and must have consented to participate. Those with comorbidity, opportunistic infection and those on other drugs outside the HAART were excluded. The control group, must be apparently healthy individuals with negative HIV screening test results who gave consent using random sampling while those who refused to give consent were excluded

### 2.5. Ethical considerations

Ethical approval for the study was obtained from Research and Ethical Committee of the institution (AEFUTHA) with approval number **AE- FUTHA/ REC/VOL 3/2020/048.**

### 2.6. Laboratory investigations

Standard methods were used for the test analysis. The HIV test was done using HIV rapid test kits (Alero Determine HIV-1/2 as first line, Unigold as second line and Chembio HIV 1/2 Stat- Pak as third line tie breaker for discordant results) following the standard algorithm. CD4 + count was done using partec cyflow analyzer, while the Viral load assay was done using Roche COBAS TaqMan 48 analyzer molecular systems. The suppression is interpreted at a viral load value of < 1000 copies per ml of plasma whereas the undetectable viral load (TND) is interpreted as values under 40–75 copies per ml of plasma (CDC). Full Blood Count determination was by the use of hematology Sysmex XE-2100 analyzer, while the detection of antibody to HAART was by Petz and direct Coombs test methods of Dacie and Lewis.

### 2.7. Data analysis

Statistical Package for Social Sciences (version 23.0) was used for data analysis. The variables were expressed as mean ± standard deviation and percentage (%). Independent *t*-test was used to compere variables with 2 means while analysis of variance was used for variables with more than 2 means. All reported *P* values are 2-tailed, and statistical significance was set at 0.05 level.

## 3. Result

**Figure 1.**
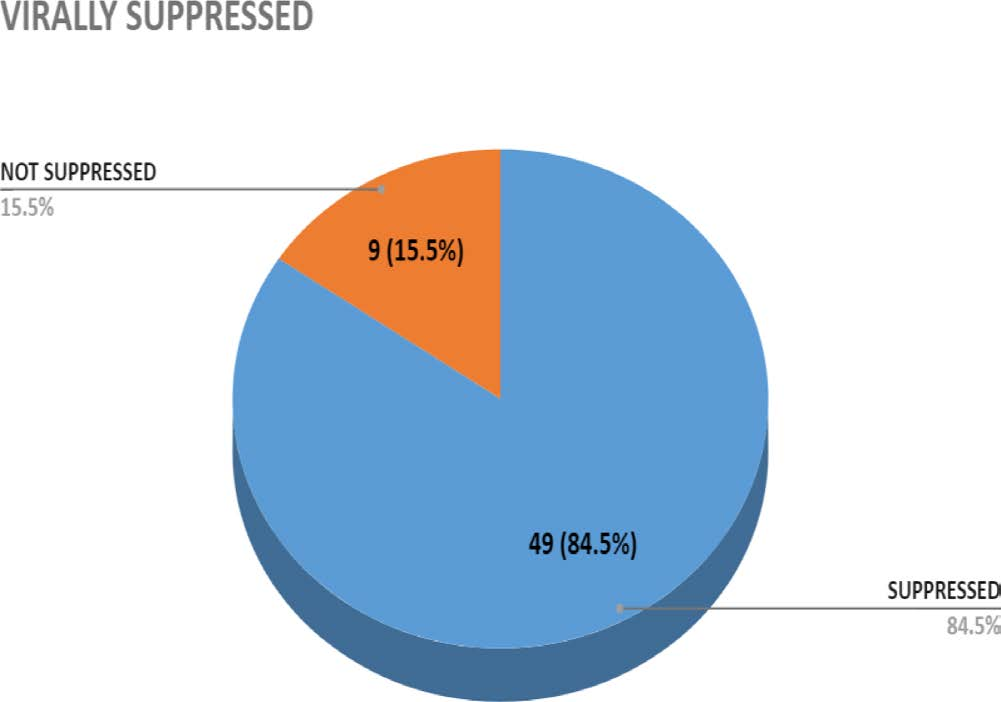
Distribution of suppression status of PLWHIV infection. PLWHIV = people living with HIV infection.

The demographics of the study population was presented in Table 1. A total of 122 subjects participated in the study consisting of 58 (48%) PLWHIV and 64 (52%) control subjects with mean age of 45.6 ± 11.5 and 38.3 ± 13.9 years respectively. Majority of PLWHIV were placed on TLD (60%) and only 84.5% of the PLWHIV have their viral load suppressed. All the PLWHIV who were recruited for the study tested positive on re-screening for HIV and no presence of circulating antibody to HAART was observed among them. Table 2 showed mean values of hemoglobin (Hb), total white cell count, Lymphocytes, Monocytes and CD4 + counts of PLWHIV to be significantly (*P* = .0001) lower than the control subjects. Three different drug regimens of HAART were used by the participants which include TLD, tenofovir/lamivudine/efavirenz(TDF/3TC/EFV),and tenofovir/lamivudine/lopinavir/ritonavir(TDF/3TC/LPV/R). The effect of the regimens on the hematological parameters as well as the viral load and CD4 count were assessed (Table 3). The Hb levels of PLWHIV on Efavirenz (123 ± 32g/l), Ritonavir (136 ± 16g/l) and dolutegravir (TLD) (134 ± 20.0g/)l indicated comparable values. The total white cell count of those on Efavirenz (4.55 ± 1.99 × 10^9^/L) and DTG (4.53 ± 1.31 × 10^9^/L) are comparable while those on Ritonavir (4.09 ± 1.15 × 10^9^/L) was significantly lower. On the other hand, Viral Load of PLWHIV on DTG (171.57 ± 4.56 copies/mml) was significantly suppressed and lower compared with the values recorded for those on Efavirenz (86395.91 ± 274762.57copies/mm) and (81188.83 ± 13393.56 copies/mml) Ritonavir respectively. However, DTG revealed significantly higher CD4 + count 713.00 ± 18.74 cell/ml than Efavirenz 556.08 ± 236.06 cell/mL and Ritonavir 518.73 ± 273.48 cell/mL. Negative direct Coombs Test (DCT) results was observed among PLWHIV (Table 4). Figure 1 showed the effect of drug regimen on the CD4 + and viral load. The bar charts indicated least suppression of HIV virus with Efavirenz but greater suppression with DTG while CD4 + count was uniformly distributed irrespective of the drug regimen they were placed on. Table 5 shows Direct Coombs Test (DCT) on PLWHIV on Drugs for Over Six (6) Months.

**Table 1:** Demographics of the study population.

Parameters		PLWHIV (n = 58)	Control (n = 64)
HIV status		Reactive	Non-reactive
Direct Coomb test		Negative	
Age		45.60 ± 11.51	38.30 ± 13.88
Gender	Male	27 (46%)	38 (60%)
	Female	31 (54%)	26 (40%)
Types of drug combination	TDF/3TC/EFV	11 (19%)	
	TDF/3TC/LPV/R	12 (21%)	
	TLD	35 (60%)	
Suppression status	Not Suppressed	9 (16%)	
	Suppressed	48 (84%)	

PLWHIV = people living with HIV infection, TLD = tenofovir/lamivudine/dolutegravir.

**Table 2:** Comparison of haematological parameters, viral load and cd4 counts on study population.

Variables	PLWHIV (n = 58)	Control (n = 64)	*t*-test	*P* value
PCV l/l	.400 ± .059	.385 ± .042	1.76	.100
Haemoglobin (g/l)	12.85 ± 1.42	13.61 ± 1.96		.037
Platelet (*10^9/L)	198.79 ± 65.08	210.64 ± 74.88	928	.355
TWBC (*10^9/L)	4.45 ± 1.41	5.44 ± 1.52	3.73	.000*
Neutrophil (abs)	2.4686 ± 1.06652	2.4205 ± 1.06892	.249	.804
Lymphocytes (abs)	4.0217 ± 16.31191	2.2205 ± .69617	.883	.379
Monocytes (abs)	.1512 ± .31635	.5159 ± .19295	7.76	.000*
Eosinophils (abs)	.0981 ± .20791	.2478 ± .36636	2.80	.006*
Viral Load	18168.72 ± 119990.74	0.00 ± 0.00	1.15	.254
CD4 + (T Cells/mL)	643.69 ± 257.08	763.81 ± 169.89	3.01	.003*

Values are represented as mean ± SD, where SD is Standard deviation.

CD4 = cluster of differentiation 4, PLWHIV = people living with HIV infection, TWBC = total white cell count.

* Significant at *P* < .05.

**Table 3:** Effect of drug combination on studied parameters of PLWHIV.

Variables	TDF/3TC/EFV(n = 11)	TDF/3TC/LPV/R(n = 12)	TLD(n = 35)	*P* value
PCV l/l	0.370 ± 0.096	0.407 ± 0.049	0.409 ± 0.044	.155
Hb(g/l)	123 ± 32.0	136 ± 16.0	134 ± 20.0	.307
Platelet × 10^9^/L	209.45 ± 74.51	208.00 ± 57.54	192.29 ± 65.47	.650
TWBC(x 10^9^/L)	4.55 ± 1.99^a^	4.09 ± 1.15^ab^	4.53 ± 1.31^bc^	.045*
Neutrophil × 10^9^/L	2.47 ± 1.77	2.56 ± 0.63	2.44 ± 0.92	.934
Lymphocyte × 10^9^/L	2.88 ± 0.52	2.02 ± 0.82	1.92 ± 0.62	.135
Monocytes × 10^9^/L	0.08 ± 0.06	0.12 ± .07	0.18 ± 0.40	.622
Eosinophils × 10^9^/L	0.07 ± 0.07	0.16 ± 0.01	0.08 ± 0.02	.464
Viral Load copies/mL	86395.91 ± 274762.57^ac^	8118.83 ± 13393.47^ab^	171.57 ± 4.56^bc^	.001*
CD4 + (T Cells/mL)	556.08 ± 236.06^ac^	518.73 ± 273.48^b^	713.00 ± 18.74^c^	.035*

Values are represented as mean ± SD,where SD is Standard deviation

* Significant at *P* < .05 Differences determined by Independent students *t*-test.

CD4 = cluster of differentiation 4, Hb = hemoglobin, PLWHIV = people living with HIV infection, TLD = tenofovir/lamivudine/dolutegravir, TWBC = total white cell count.

**Table 4:** HIV rescreening status of the PLWHIV.

HIV RESCREENING	PLWHIV	Control
Reactive	58 (100%)	0 (0%)
Non-reactive	0 (0%)	64 (100%)
Grand total	58 (100%)	64 (100%)

PLWHIV = people living with HIV infection.

**Table 5:** Direct coombs test (DCT) on PLWHIV on drugs for over six (6) mo.

Duration of enrollment	No. enrolled	DCT result
6 mo - 5 yr	8 (14%)	NEGATIVE
6 yr - 10 yr	32 (55%)	NEGATIVE
11 yr - 15 yr	18 (31%)	NEGATIVE
Total	58 (100%)	NEGATIVE

PLWHIV = people living with HIV infection.

## 4. Discussion

This study was conducted to provide information on the impact of the new introduced highly active antiretroviral therapy on hematological parameters, viral load, CD4 count, HIV re-screening status and antibody to HIV antiretroviral protocol among people living with HIV infection attending clinic at Alex Ekwueme Federal Teaching Hospital Abakaliki, Ebonyi State. The Current state of practice in HIV treatment is commencement of HAART upon testing positive on screening, obtaining the viral load baseline and repeat after 6 months for monitoring. By this practice, HIV subjects naïve to HAART no longer exist and the use of CD4 count as a tool for establishing HAART initiation is also no longer in use. The 58 HIV infected patients on HAART had a mean age of 45.6 ± 11.51years, while their gender distribution showed 54% females and 46% males. The control group had 64 non-infected subjects drawn from the general population comprising of 41% females and 59% males with the mean age of 38.3 ± 13.88 years. Age and gender differences were observed. The disparity in the percentage distribution of the female and male participants seen in the study may be attributed to the willingness of the individuals to be involved. This pattern of finding has been reported in previous research.^[14]^ The HIV status of the PLWHIV remained positive irrespective of being on HAART. This finding has revealed that HIV status cannot change over time with treatment rather the viral load can go down to undetectable level which will significantly reduce the transmission risk of HIV. Viral load suppression was also observed. The suppression rate of 84.5% is at par with the 86% national rate reported for Nigeria^[11]^ but higher than that reported by Abudiore et al, 2023 75.71%.^[12]^ The higher percentage obtained may be due to the small sample size used. Antibody screening for the presences of antiretroviral drug antibody was performed using Petz and direct Coombs test method and all the participants tested negative irrespective of the duration of the treatment.^[13,14]^ This disagrees with previous work of Omoregie *et al*,^[15]^ where a total of 81 (81%) out of the 100 HIV patients on HAART had antibodies to 1 or more of the ART drugs. The reason for the disparity may be attributed to the type of ART drug used. While Omoregi et al used subject on momo ART, the current study accessed subjects on highly active retro-viral drugs (Combined therapy). Moreso, some of the drugs used as at than (such as Stavudine, Nevirapine, Zidovudine etc) have since been discontinued.

Infection with HIV has been associated with a broad range of clinical outcomes involving the hematopoietic system. Hematological complications have been documented to be the second most common cause of morbidity and mortality in HIV patients, and are generally marked with cytopenia such as anemia, neutropenia, lymphopenia and thrombocytopenia.^[16]^ The Packed cell volume of PLWHIV 0.401 ± 0.06 L/L is comparable with the control group 0.386 ± 0.04L/L.(*P* > .05) while, Hb level was (128 ± 14.2g/l) significantly lower (*P* = .002) than that of the control group (136.1 ± 19.6g/l). This is in agreement with the findings of previous researchers who reported anemia. The lower Hb level in PLWHIV implies anemia and the possible reasons include poor utilization of iron, ineffective erythropoiesis and possible red cell destruction as reported in previous studies.^[17,18]^ Neutropenia is the most common leucopenia occurring in HIV infected individuals. HIV infection on its own suppresses the bone marrow and leads to decreased levels of granulocyte colony-stimulating factor, and also affects the granulocyte-macrophage lineage, thus resulting in leukopenia and neutropenia.^[19]^ The findings of the current study revealed comparable neutrophil, monocyte, lymphocyte and platelet counts with the control group thus, indicating the efficacy of `the current HAART regimen. CD4 immunodeficiency and increased viral load has been noted as the hallmark of HIV infection, with leucopenia and lymphopenia being documented in different proportions in HIV-positive patients.^[19]^^[Bibr R19]^ The CD4 count normal range is between 500 and 1500 cell/mm and when left untreated, levels can drop below 200 cell/mm which is one indication for diagnosis of AIDS. Again, CD4 is used to evaluate the progress of HIV and the result obtained can offer insight in to the management of HIV, the risk of opportunistic infection as well as an indicator of treatment failure.^[11]^ The CD4 + count of PLWHIV were significantly (*P* = .0001) lower (643.69 ± 257.08 cell/mm^3^) than the control subjects (763.81 ± 169.89 cell/mm^3^) however the finding revealed higher CD4 Count levels among the study subjects indicating good management by the center.

**Figure 2.**
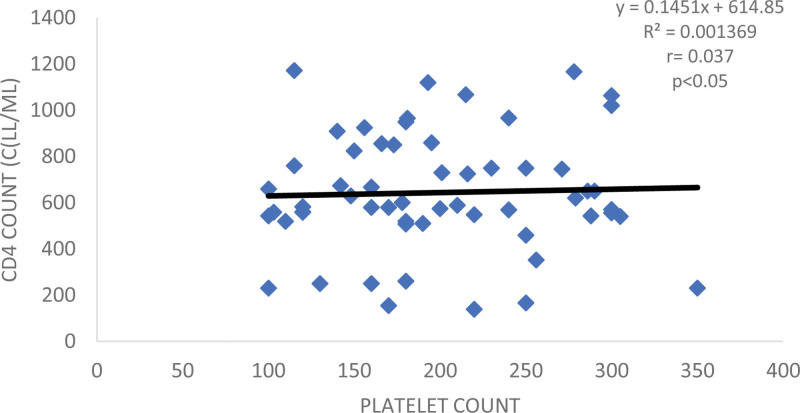
Effect of drug type on CD4 + and viral load. CD4 = cluster of differentiation 4.

**Figure 3.**
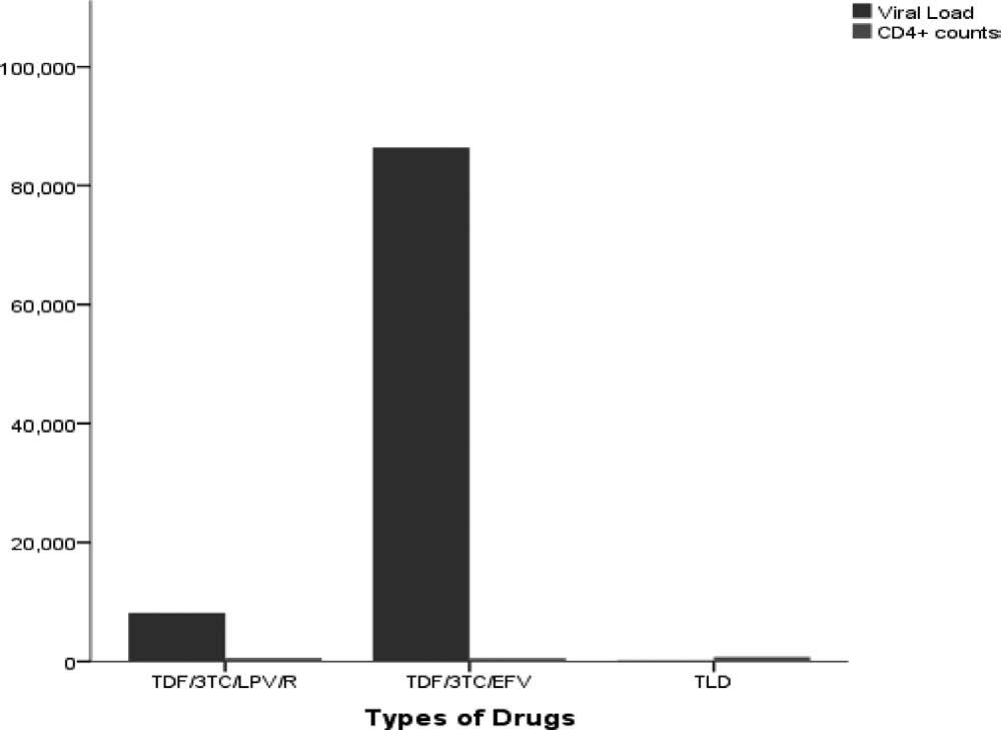
Scatter plot showing the relationship between CD4 + counts and PLT count. CD4 = cluster of differentiation 4.

The 3 regimens used by the participants (DTG, Efavirenz, and Ritonavir) revealed comparable packed cell volume (PCV) and Hb levels. Efavirenz and DTG had comparable total white cell count values while Ritonavir had significantly lower count when compared with Efavirenz and DTG respectively. Furthermore, DTG provided a very good suppression though from a small sample size is in line with the known property of DTG in achieving rapid viral suppression, thus supporting the use of DTG as preferred first line regimen. This finding is supported by previous reports that DTG is a highly effective inhibitor-DNA integrase that prevents the HIV from integrating into the host cell through blocking of the strand transfer step of retroviral-DNA which is essential for HIV replication cycle thus, making it to have a high genetic barrier to developing drug-resistance. Efavirenz on the other hand, failed viral load suppression and is attributed to the low genetic barrier to the development of drug-resistance viral mutants.^[20]^ Finding also revealed improvement on the CD4 + count by DTG and it is understandably so because of the viral reduction associated with it. Figure 2, affirms the above finding. The limitation of the current study was the unwillingness of the subjects to participate and obtaining those on treatment adherence hence the small sample size of 58. Therefore, the information may not be a true reflection of the entire state. More work is suggested involving larger number to include all the treatment sites in Ebonyi State as well as collaboration with other states in Southern Nigeria. Figure 3 showed scatter plot showing the relationship between CD4 + counts and PLT count.

## 5. Conclusion

Some hematological parameters in PLWHIV are comparable with that of the control group. Within the 3 regimens, DTG provided better option as first line regimen as against the other 2 due to its extent of viral suppression and improved CD4 + count whereas Efavirenz failed viral load suppression.

## Author contributions

**Conceptualization:** Stella N. Echefu.

**Data curation:** Stella N. Echefu.

**Formal analysis:** Euphoria C. Akwiwu.

**Investigation:** Joseph E. Udosen, Emmanuel Ifeanyi Obeagu.

**Methodology:** Emmanuel Ifeanyi Obeagu.

**Project administration:** Euphoria C. Akwiwu.

**Supervision:** Josephine O. Akpotuzor.

**Writing – original draft:** Emmanuel Ifeanyi Obeagu.

**Writing – review & editing:** Emmanuel Ifeanyi Obeagu.
